# Obesity and COVID-19 in Children and Adolescents: Reciprocal Detrimental Influence—Systematic Literature Review and Meta-Analysis

**DOI:** 10.3390/ijerph19137603

**Published:** 2022-06-21

**Authors:** Giusy La Fauci, Marco Montalti, Zeno Di Valerio, Davide Gori, Maria Giulia Salomoni, Aurelia Salussolia, Giorgia Soldà, Federica Guaraldi

**Affiliations:** 1 Unit of Hygiene, Department of Biomedical and Neuromotor Sciences, Public Health and Medical Statistics, University of Bologna, 40126 Bologna, Italy; giusy.lafauci@studio.unibo.it (G.L.F.); marco.montalti7@studio.unibo.it (M.M.); zeno.divalerio@studio.unibo.it (Z.D.V.); mariagiulia.salomoni@studio.unibo.it (M.G.S.); aurelia.salussolia@studio.unibo.it (A.S.); giorgia.solda@studio.unibo.it (G.S.); 2 IRCCS Istituto delle Scienze Neurologiche di Bologna, 40126 Bologna, Italy; federica.guaraldi@yahoo.it

**Keywords:** pediatric obesity, eating habits, physical activity, lockdown, COVID-19, disease severity, meta-analysis

## Abstract

The dramatic lifestyle changes forced by COVID-19-related lockdown promoted weight gain, with a stronger impact on obese subjects, at higher risk of severe infection. The PubMed database was searched to identify original studies assessing: (1) the extent and risk factors of lockdown-induced weight increase; and (2) the impact of obesity on the risk of hospital admission in children and adolescents. A systematic literature review and meta-analyses were performed. Twenty out of 13,986 identified records were included. A significant weight increase was reported in the majority of subjects, with no apparent gender or age differences. It was induced by a higher consumption of hypercaloric/hyperglycemic/junk food and/or the reduction of physical activity, often associated with an altered sleep–wake cycle. On the other hand, obesity increased the risk of hospitalization (OR = 4.38; 95% C.I. 1.46–13.19; *p* = 0.009; *I*^2^ = 96%) as compared to the normal weight population. COVID-19 and obesity represent epidemic conditions with reciprocal detrimental impact. Urgent public health interventions, targeting the various age and social strata, and involving governmental authorities, health care personnel, teachers and families are warranted to increase awareness and actively promote healthy lifestyles to contrast pediatric obesity and its detrimental consequences at a global level.

## 1. Introduction

COVID-19, caused by SARS-CoV-2 infection and characterized by acute respiratory syndrome with high morbidity and mortality, was first discovered in Wuhan (China) in December 2019 and rapidly spread all over the world, becoming a pandemic [1,2].

To limit its diffusion, most of the countries adopted unprecedented restriction measures to ensure social distancing, including home quarantines and national closure of schools, working sites and other public places [3]. The generalized lockdown forced the entire population to sudden and drastic lifestyle changes and increased the perceived stress and anxiety associated with the pandemic [3].

Overall, caloric intake significantly increased, while physical activity was drastically reduced. A recent meta-analysis showed significant increases in body weight and BMI during closure among school-age children and adolescents, as well as the increase of obesity and overweight prevalence [4]. Moreover, the sleep–wake cycle was altered, with longer day sleep time and night insomnia, the latter contributing to night eating. Their combination promoted weight increase (“obesogenic environment”) in adults and children [5,6].

Since the early 2000s, pediatric obesity has been recognized as an alarming global health issue of increasing proportions, induced by unhealthy eating habits, sedentary lifestyle, stress and psychological disorders [7,8]. To underline the direct detrimental effect of COVID-19 lockdown on obesity, the term ‘*covibesity*’ has been introduced [9].

Contextually, obesity has been demonstrated as an independent risk factor of more severe COVID-19 disease and worse prognosis in children [10], who otherwise present with mild symptoms [11,12,13,14,15,16,17].

Based on these premises, we aimed to systematically and critically analyze: (1) the incidence and potential predictors of obesity induced by COVID-19 pandemic-lockdown; and (2) the risk of severe COVID-19 disease in obese children and adolescents.

## 2. Materials and Methods

The PubMed database (https://pubmed.ncbi.nlm.nih.gov, accessed on 31 December 2021) was searched for studies on COVID-19 in children and adolescents using the purposely formulated string: “(SARS-CoV-2 OR COVID-19 OR ncov* or coronavirus) AND (child OR children OR pediatric OR paediatric OR infant OR adolescent)”. Only original studies (i.e., cohort, cross-sectional and case-control) including patients aged 0–18 years of both genders, written in English or in Italian, published from December 2019 to December 2021, for which the abstract was available, were kept.

Reviewer pairs (G.L.F. and M.M.; Z.D.V. and M.G.S; A.S. and G.S.) were created. Each reviewer independently screened articles by title and abstract to identify eligible studies. Any disagreement was solved through discussion among the reviewers and team consensus. The same authors evaluated the full texts of relevant studies. For each full text, the following information was recorded: title, author(s), country, study period (months), study design, sample size, population, age (mean ± SD and range) and gender. To determine the impact of lockdown on weight, we also recorded baseline weight/BMI, weight/BMI increase, modifications of eating habits and/or physical activity. Finally, we recorded information on sleep disorders/modification of sleep–wake habits. On the other hand, to assess the impact of overweight/obesity on hospital admission, we recorded information on the number of obese children/adolescents included in the sample, the number of obese hospitalized and the number of normal weight children/adolescents hospitalized.

We tested statistical heterogeneity to determine if it was appropriate to combine the studies for meta-analysis. We analyzed statistical heterogeneity to test the robustness of matching the studies for meta-analysis, evaluating heterogeneity by the use of graphic forest plots and by calculating the *I*^2^ statistic, which represents the percentage of the variance in effect estimates that is caused by heterogeneity rather than by sampling bias (chance). We considered an *I*^2^ statistic greater than 40% to be substantially heterogeneous. According to the *Cochrane Handbook for Systematic Reviews of Interventions* [18], if studies were <5 or substantially heterogeneous, we used a random-effects model, following the method of DerSimonian and Laird, to compute the random-effects estimates for the corresponding statistics [19]. Forest plots were created to display effect estimates with 95% CIs. For all data analyses, the RevMan program was used (Version 5.4.1; The Nordic Cochrane Centre, The Cochrane Collaboration, Copenhagen, Denmark, 2014) [20].

## 3. Results

The database search retrieved 13,986 articles, including 13,845 from the PubMed search, 28 from a bibliographic search of relevant papers, and 113 from hand search. Sixty-seven were excluded as they were duplicates, while 13,752 were excluded because they were not pertinent with the review aims. Of the remaining 167,147 records were excluded because they were not original studies, or did not focus on the topic of interest, i.e., did not evaluate the impact of lockdown on obesity, or did not report the outcome of COVID-19 specifically in children or adolescents.

**Figure 1:**
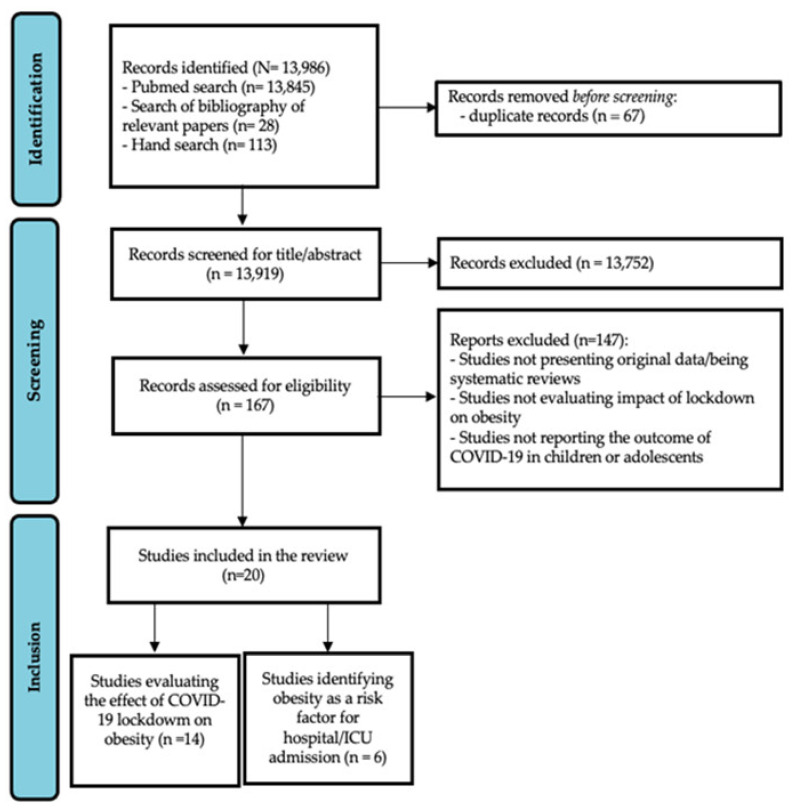
PRISMA flow chart showing the study selection process.

Finally, 20 articles were considered eligible for full-text evaluation: 14 evaluated the effect of COVID-19 lockdown on obesity (first review aim), while six focused on obesity as a risk factor for hospital/Intensive Care Unit (ICU) admission (second review aim). The PRISMA flowchart, showing the study selection process, is reported in Figure 1.

### 3.1. Impact of COVID-19 Lockdown on Weight and Lifestyle in Children and Adolescents

Relevant data of the 14 studies focusing on the impact of COVID-19 lockdown on changes in weight and lifestyle in children and adolescents are summarized in Table 1.

Sample size ranged from 40 [31] to 274,456 [32] patients. Males represented 24 (678/2824) [26] to 77.8% (70/90) [28] of the study population. Age ranged from 0 [32] to 18 years [32,33], and mean age ± SD from 7.8 ± 4.1 [21] to 17.5 ± 1.2 [26]. Ten studies were performed in hospitals (71.4%), 2 (14.3%) online/via social-media, and 2 (14.3%) in schools.

Eight (57.1%) studies evaluated changes of patients BMI-SDS/z-score [22,23,25,27,28,29,33,34], six (42.9%) of Body Mass Index (BMI) [24,26,28,29,30,31] and four (28.6%) [21,26,28,29] of patient weight. Vinker-Shuster M et al. reported adjusted weight percentiles [32]; Maltoni et al. reported modifications also in waist circumferences and waist/height ratio [29].

At baseline evaluation, performed before COVID-19 lockdown, BMI ranged from 20.9 (median BMI) [30] to 32.6 ± 4.0 (mean ± SD) [29]; BMI-SDS/z-score ranged from 0.001 (median BMI-SDS) [33] to 2.4 ± 0.5 (mean ± SD) [29], and mean body weight from 32.3 ± 16.9 [21] to 67.2 ± 23.8 [28].

At follow-up evaluation, performed at the end of the lock-down, six studies (42.9%) reported the increase of BMI [25,26,28,29,30,31], 5 (35.7%) of body weight [21,26,28,29,32], and five others of BMI SDS/z-score [23,28,29,33,34]. According to four others (14.3%) weight distribution remained stable [21,22,24,27], although the last two evaluated only a part of the study population.

Three (21.4%) studies reported ‘changes in eating habits’ associated with lockdown that consisted of a significant increase of the intake of fresh fruit, vegetables, dairy products, pasta, sweets (especially at breakfast) and snacks in the study by Androutsos et al. [21], and of pasta, bread and pizza according to Cipolla et al. [24], and in the decrease of vegetable/fruit intake in the study of Maltoni et al. [29]. Valenzise et al. [31] reported an overall increase in BMI during lockdown, although not significant, associated with an overall increase in the number of daily meals, especially in children with parents with elementary school diploma vs. high school diploma (6 ± 0.7 vs. 4.4 ± 1.3; *p* = 0.019).

Six (42.9%) studies reported a reduction of physical activity [21,24,26,28,29,31], together with an increase in video-gaming [24], while two (14.3%) focused on the impact of COVID-19 lockdown on sleep hygiene and demonstrated an overall increase of sleep hours [21,26]. Azoulay et al. [22] reported the positive effect on body composition associated with engagement in physical activity during the lock-down, although data on lifestyle changes (type and prevalence) were not reported.

According to Brooks et al. [23] and Hu et al. [25], children aged 8–12-years old and 6–11-years old, respectively, had a more marked increase in weight gain than adolescents. Moreover, four studies reported a higher weight increase in boys than in girls in [23,25,29,30], likely related to a more significant increase of sedentary behavior, electronic gaming and screen time, especially in younger children.

Pre-existing weight excess/obesity [23], lower socioeconomic position, a lack of health insurance/Medicaid, Black and Hispanic ethnicity increased the risk of weight increase [22,23]. In the study by Hu et al. [25], no significant differences in BMI increase associated with lockdown were detected in children living in urban and rural areas.

### 3.2. Impact of Overweight/Obesity on the Risk of Hospital/ICU Admission in Children/Adolescents with COVID-19

The main data of patients enrolled in the six studies [35,36,37,38,39,40] assessing the impact of overweight/obesity on hospital/ICU admission in children/adolescents with COVID-19 are shown in Table 2.

Sample size ranged from 48 [40] to 30,527 [39] patients. All studies included both children and adolescents. Males represented 45.6 [37] to 63% [40] of the sample. Prevalence of obesity varied from 2.8 [39] to 39.6% [40]; all obese patients presented with COVID-19 symptoms.

The rate of hospital admission among obese patients ranged from 31.3 [37] to 60.3% [36], and from 9.3% [38] to 24.8% [37] in normal weight ones. In three studies, all patients recruited were admitted to hospital/ICU [35,39,40].

Obesity was found to be an independent risk factor for severe/critical illness in two (33.3%) studies [35,37] and for hospital admission in three (50%) studies [36,38,40].

Other risk factors associated with more severe COVID-19 with hospital/ICU admission were adolescence [37,39], age < 1 year [39], Type 1 diabetes [38] and black ethnicity [39].

**Figure 2:**
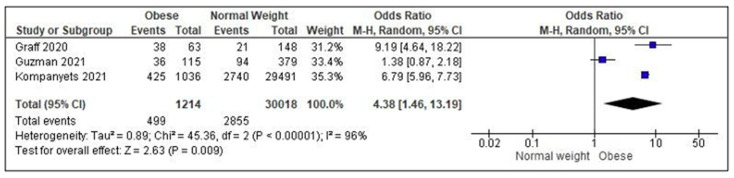
Meta-analysis assessing the risk of hospitalization/admission to Intensive Care Units in obese children/adolescents as compared to normal weight peers. Studies are listed in alphabetical order [36,37,38].

Due to the wide heterogeneity of outcomes, investigation tools and statistical analysis, only three studies could be included in the meta-analysis [36,37,38], showing a significantly increased risk of hospitalization for obese children/adolescents as compared to normal weight peers (OR = 4.38; 95% C.I. = 1.46–13.19; *p* = 0.009; *I*^2^ = 96%) (Figure 2).

## 4. Discussion

The aims of the present systematic literature review and meta-analysis were to assess in children and adolescents the impact of COVID-19 lockdown in promoting weight gain and obesity, on one hand, and the risk of developing severe COVID-19 disease, with consequent admission to hospital/ICU, associated with overweight/obesity on the other hand.

COVID-19 and pediatric obesity can be approached as “*syndemic conditions*”. This notion, conceived by the medical anthropologist Merrill Singer in the 1990s, goes beyond comorbidities as it identifies conditions characterized by biological and social interactions that increase a person’s susceptibility to harm or worsen health outcomes, so are important for prognosis, treatment and health policy. As the same author explained, a “syndemic approach” provides a very different orientation to clinical medicine and public health as an integrated approach to understanding and treating diseases can be significantly more successful than simply controlling epidemic disease or treating individual patients [41]. In 2020, Horton suggested applying the term ‘syndemic’ to COVID-19 for its clustering and interactions with pre-existing conditions, and the influence of larger political, economic and social factors [42].

### 4.1. Impact of COVID-19 Lockdown on Weight and Lifestyle Changes in Children and Adolescents

Data analysis clearly demonstrated the detrimental impact of COVID-19 lockdown on children and adolescents’ body weight and BMI, children with pre-existing overweight/obesity being more at risk of gaining weight.

Obesity is a chronic disease resulting from the interaction of genetic, environmental, and psychosocial factors leading to the predominance of caloric intake over expenditure. Based on the standardized growth charts of the Centers for Disease Control and Prevention [43], youths are defined as ‘overweight’ for body weight in the 85th–94th centile, ‘obese’ in the 95th–98th centile, and ‘severely obese’ if >99th centile. Even before the COVID-19 pandemic, pediatric weight excess was considered a worrisome epidemic condition affecting over 337 million children globally, with more than 124 million cases of obesity and severe obesity, worsening over the time, with rates varying with age, ethnicity, location, and social determinants [7]. The associated health and social burden depend on the several physical and psychological co-morbidities, characterized by early onset and lifetime duration, with an overall poor patient quality of life and significant social costs [44,45,46].

Weight gain seems to be explained primarily by the increase of sedentary life, secondary to the longer time spent at home sitting and screen time for homeschooling and recreational activities (i.e., video games, computers/tablets, and television), in the absence of a structured environment on weekdays, to the detriment of compulsory physical activity at school and extracurricular physical activity in dedicated recreational spaces and outdoors [21,26,27,29,32,39]. This phenomenon appeared to be more prevalent among younger children with respect to adolescents, and in boys as compared to females [23,25,29,30]. As demonstrated by previous studies, the abrupt cessation of exercise and prolonged inactivity promote several other adverse health changes, including insulin resistance, muscle atrophy and bone loss [47].

Change in dietary habits towards unhealthy patterns and lifestyles characterized by an overall increase of ingested calories, secondary to a higher number of meals per day [31], a more abundant breakfast [21], and the consumption of hypercaloric food at the various meals (i.e., sweets, snacks, carbohydrates, junk food [21,24,29]), significantly contributed to weight increase during COVID-19 lockdown.

Some studies also reported the alteration of sleep-wake cycles during the lockdown period—predisposing to night eating [26,27,32] and altering the hormone circadian rhythms—[48,49] and the overall increase of sleep time, reducing hours dedicated to physical activity and, thus, further contributing to the sedentary lifestyle [21,26].

Finally, the high levels of stress, fear and anxiety experienced by children and adolescents during COVID-19 pandemic could have contributed to weight increase during the lockdown [26,28]. Previous studies had demonstrated the complex relationship between stress, mental health, and obesity [50]. First, stress can cause weight gain by stimulating chronic cortisol secretion [51,52]. Second, prolonged stress periods can lead to depression with consequent isolation, home stay, sedentary behavior, and unhealthy nutrition, overall contributing to weight increase and obesity, that are, in turn, responsible for social stigma, stress and isolation [50]. Factors promoting stress and anxiety in children and adolescents were self-experienced but also parent-transmitted and included poor knowledge (especially at the beginning of the pandemic) of SARS-CoV-2 mechanisms of transmission, potentially severe outcomes of infection, and absence of efficacious treatments, social isolation, drastic changes in family dynamics and exacerbation of dysfunctional aspects, economic difficulties secondary to job loss/unemployment and difficulties in the use of technology necessary for home schooling and working [53,54].

Interestingly, the significant increase of body weight and BMI reported by the various studies occurred in a very short period, confirming previous observations on higher weight gain during summer vacations than during the school year, thus reinforcing the importance of school in obesity prevention through structured routine with meals, physical activity and a routine that promotes an adequate sleep schedule [55,56]. Forced school closure during the COVID-19 pandemic had a greater impact because of the previously mentioned associated psychological aspects impairing mental health and well-being, and the detrimental effect on academic progression [50].

The highest weight/BMI increase was observed in children with pre-existing overweight/obesity, of Hispanic and African American ethnicity, and in those living unfavorable socioeconomic conditions, therefore, most vulnerable to unhealthy lifestyle, food insecurity, family and social stress (i.e., lower parental psychological and educational support, and higher financial concerns/limitations), and with difficult access to academic resources and healthcare services [22,23,50].

### 4.2. Impact of Overweight/Obesity on the Severity and Outcome of COVID-19 Disease

Our study clearly pointed out a significant increase in the risk of severe COVID-19 disease and, consequently, of hospital/ICU admission in youths with overweight/obesity as compared with normoweight pairs [35,36,40,57].

The prevalence and severity of COVID-19 disease in children is significantly lower than in adults. It seems that, because of their immune systems’ immaturity, younger individuals are less likely to develop severe disease than adults and are more often asymptomatic. Most of the cases occurred in children aged 5–10 years old, and the prevalence in males was slightly higher than in females [58,59].

Around 3% developed critical conditions and few died. Racial/ethnic minorities and low-income populations, as well as children with comorbidities (i.e., chronic cardiac, respiratory, kidney, oncological, immunological, and hematological diseases, especially if associated with immunosuppression; diabetes and obesity) presented a significantly higher risk of infection and adverse disease outcome [60,61].

It should be underlined that obesity appeared to be the most significant independent risk factor, even in the mildest cases, in agreement with data from the adult population [12,14,16,61]. Several altered mechanisms, related to obesity itself and to its comorbidities, are involved. These include: (1) *altered respiratory physiology*, secondary to the pressure exerted by abdominal adiposity on the lungs, and the *defective lung mesenchymal stem cells*, responsible for ineffective tissue repair processes and immune response, with an overall increased risk of pulmonary infections, asthma, and obstructive apneas; (2) *insulin resistance* and *hyperinsulinemia*, because of which, in situations of intense metabolic activity, like during the response to SARS-CoV-2 infection, beta cells, already working at their limit, were not able to increase insulin secretion and can also be damaged by the virus [62,63]. Moreover, they contribute to the onset of other metabolic and cardiovascular alterations; including (3) *dyslipidemia* (low HDL- and increased LDL-cholesterol levels contribute to endothelial dysfunction and atherosclerosis); (4) *hypertension* with consequent left ventricular hypertrophy; (5) *non-alcoholic steatohepatitis*; and (6) *hyperuricemia* [62,63]. Finally, insulin resistance increases the (7) *oxidative stress* and, together with visceral adiposity, endothelium damage and micronutrients deficiencies (i.e., vitamin D, C, A, E and B12, iron and folate, with anti-oxidative action) lead to (8) *chronic inflammation*, *excessive and dysregulated inflammatory response*, and *increased coagulation activity* [62,63].

### 4.3. Study Strengths and Limitations

The main strengths of this work are: (1) the ‘syndemic’ approach to pediatric obesity and COVID-19 disease, according to which their reciprocal influence has been evaluated by dedicated analyses in the same study; (2) the accurate study search—through the initial application of a purposely-created highly sensitive string to identify as many pertinent articles as possible, so reducing the risk of missing important data—and selection; (3) the inclusion of studies focusing only on children and adolescents, thus discarding those including adults together with youths, thus increasing the specificity of the study.

On the other hand, the study presents some important limitations. First, the limited number and heterogeneity of the studies, in terms of sample size, gender distribution, ethnicity, study design and parameters used for the assessment of study variables, outcomes and confounders could be only partially limited by the accurate selection made. Most of the studies were cross sectional and some were based on parent interviews, thus limiting the validity of collected data because of intrinsic study limitations. Moreover, some population samples may be overlapping. Finally, the majority of the studies considered absolute weight increase and/or BMI, while only few reported weight and BMI percentiles, which are fundamental to assessing the presence and degree of weight excess in children and adolescents.

## 5. Conclusions

The COVID-19 pandemic had a huge impact on the health and well-being of children and adolescents. Lockdown measures affected healthy lifestyle behaviors through the modification of dietary habits, the reduction of physical activity and the alteration of sleep patterns, and also increased the levels of stress and anxiety, overall promoting weight gain and obesity. Moreover, pediatric overweight/obesity—already representing an alarming epidemic of increasing proportions, with detrimental effects on physical and mental health before COVID-19, has been demonstrated to be the most important independent risk factor for the development of severe SARS-CoV-2 infections in youths, requiring admission to hospital/ICUs.

Urgent measures aimed at supporting children and their families through counseling and active implementation of services for the promotion of healthy lifestyle in the different settings as well as medical care at all population levels are mandatory to limit the outburst of pediatric obesity. Finally, active population surveillance, as well as structured prospective studies focusing on physical and psychological aspects, are warranted to assess the real impact of COVID-19 disease and related containment measures on youth health and wellbeing in the long-term, and, consequently, promote targeted, more efficacious Public Health interventions.

## Figures and Tables

**Table 1:** Main patient and protocol features of studies assessing the impact of COVID-19 lockdown on weight and lifestyle changes included in the literature review.

Author, Year	Country	Study Period (Months)	Study Design	N	Males (N, %)	Population	Age (Mean ± SD; Range)	Setting	Weight Measure	Weight Status Before Lockdown	Weight Status After Lockdown	Change in Weight Status	Change in Eating Habits	Decrease in Physical Activity (PA)	Sleep Changes
Androutsos O. et al., 2021 [21]	Greece	2	CSS	397	228 (57.4)	C, A	7.8 ± 4.1 *	O	BW	32.3 ± 16.9 *	n.a.	stable BW: N = 214 (58.9%); BW increase: N = 127 (35%); BW decrease: N = 22 (6.1%)	Increase in fresh fruit juices, vegetables, dairy products, pasta, sweets, snacks and breakfast	N = 261 (66.9%)	Increased sleep time (h/d). BL: >10 h/d = 13.3%, <8 h/d = 15.4% vs. AL: >10 h/d = 24.2%, <8 h/d = 4.8%
Azoulay E. et al., 2021 [22]	Israel	7	LS	220	109 (49.5)	C, A	10.8 ± 3.2 *	H	BMI-SDS	BMI-SDS: 1.74 (1.40, 2.03) **	BMI-SDS: 1.70 (1.36, 1.97) **	MFR increase in underweight (*p* = 0.05) and normal weight (*p* = 0.008), but not in overweight/obese patients. Associations in BMI z-scores (*r* = 0.961, *p* < 0.001) and MFR z-scores (*r* = 0.854, *p* < 0.001) before and during the pandemic. A multivariate linear regression model identified socioeconomic position, pre-pandemic BMI and MFR z-scores, and physical activity levels during the pandemic as predictors for delta MFR z-scores (*F* = 12.267, *p* < 0.001)	n.a.	n.a.	n.a.
Brooks C.G. et al., 2021 [23]	USA	12	HC	96,501	n.a.	C, A	6–17	H	BMI-SDS	0.31 (0.29, 0.32) **	0.62 (0.59, 0.64) **	Overall increased BMI-SDS: 0.30 (0.27–0.33) *. (In obese C AL: 1.16 (1.07–1.24) ** vs. BL: 0.56 (0.52–0.61) **; Hispanic C AL: 0.93 (0.84–1.02) ** vs. BL: 0.41 (0.36–0.46) **; C lacking commercial insurance AL: 0.88 (0.81, 0.95) ** vs.BL: 0.43 (0.39, 0.47) **; DBMI higher in boys vs. girls (0.36 vs. 0.24)	n.a.	n.a.	n.a.
Cipolla C. et al., 2021 [24]	Italy	1	CSS	64	26 (40.6)	C, A	13.9 ± 2.4 *	H	BMI	27.7 ± 4.8 *	27.6 ± 4.0 *	BMI increase: N = 31 (48.4%); BMI decrease: N = 33 (51.6%)	Increase in bread/pasta/pizza (N = 43; 67.2%); desserts (N = 3; 4.7%), meat (N = 8; 12.5); vegetables/fruit (N = 10; 15.6%); sugar drinks (N = 20; 31.2%)	Higher BMI increase in sedentary patients (*p* = 0.024) and in those spending longer time at videogaming (*p* = 0.005)	n.a.
Hu J. et al., 2021 [25]	China	12	HC	207,536	n.a	C, A	6–17	H	zBMI	0.29 ± 0.01 *	0.45 ± 0.01 *	Increase of *z*BMI and OB in 2020 vs. 2014–2019 in all age groups, but significant only for ages 6–11 and 15–16. *z*BMI increase in boys (0.18) higher than in girls (0.13, *p* = 0.014). Similar rise in urban and rural areas	n.a.	n.a.	n.a.
Jia P. et al., 2020 [26]	China	1	CSS	2824	678 (24.0)	A	17.5 ± 1.2 *	S	BW; BMI	BW: 58.6 ± 17.1 * BMI: 22.7 ± 6.7 *	BW: 60.2 ± 22.9 *; BMI: 23.6 ± 8.6 *	Increase in mean BMI and BW	n.a.	Decrease in moderate-/vigorous-intensity PA: 0.5 ± 1.7 * vs. 0.4 ± 1.7 * d/w	Increase in sleeping time: sleep (h/d): 7.5 ± 3.2 * vs. 7.7 ± 4.7 * (workdays); 8.0 ± 3.4 * vs. 8.2 ± 5.4 * (weekends)
Kang H.M. et al., 2021 [27]	South Korea	6	HC	226	96 (42.5)	C, A	10.5 (8.7–12.4) **	H	zBMI	0.4 ± 1.3 *	0.2 ± 1.3 *	OW/OB: 31.4 vs. 23.9 % (*p* = 0.074); increase from NW to OW/OB: 9.5%. Mean zBMI 0.42 ± 1.25 vs. 0.2 ± 1.25 (*p* < 0.001) Days after school closure (*p* = 0.004) and normoweight (*p* = 0.017) pre-COVID were negative predictors	n.a.	n.a.	n.a.
Kim E.S. et al., 2021 [28]	South Korea	6	HC	90	70 (77.8)	C, A	12.2 ± 3.4 *	H	BW; zBW; BMI; zBMI	BW: 67.2 ± 23.8 *; zBW: 2.0 ± 0.8 *; BMI: 26.7 ± 4.6 *; zBMI: 1.9 ± 0.5 *	BW: 71.1 ± 24.2 *; zBW: 2.2 ± 0.7 *; BMI: 27.7 ± 4.6 *; zBMI: 2.0 ± 0.4 *	△zBW: 0.18 (0.1–0.29) **; △zBMI 0.06 (0–0.12) **	n.a.	yes	n.a.
Maltoni G. et al., 2021 [29]	Italy	3	LS	51	31 (60.8)	C, A	14.7 ± 2.1 *	H	BW; BMI; BMI SDS; WC; W/H-r	BMI: 32.6 ± 4.0 *; BMI SDS: 2.4 ± 0.5 *; WC: 102.1 ± 12.6 *; W/H-r: 0.6 ± 0.1 *	n.a.	△BW: 2.8 ± 3.7 *; Δ-BMI: 0.5 ± 1.3 *; Δ-BMI SDS: 0.1 ± 0.2 *; ΔWC.: 4.4 ± 7.8 *; ΔW/H-r: 0.02 ± 0.005 * △BW: M 3.8 ± 3.4 vs. F 1.2 ± 3.7 (*p* = 0.02) Δsedentary behavior: M3.8 ± 2.7 vs. F 1.5 ± 2.5 (*p* = 0.003)	Δ-intake of vegetables/fruit: −0.1 ± 0.5 * (portions/w)	yes	n.a.
Qiu N. et al., 2021 [30]	China	7	LS	446	260 (58.2)	C	7–12	S	Median BMI	20.9 kg/m^2^	22.4 kg/m^2^	Increase from NW to OW/OB in 28.1%; from OW to OB in 42.42%. Boys at significantly higher risk	Increased number of meals, higher in parents with primary school vs. high school diploma (6 ± 0.7 vs. 4.4 ± 1.3, *p* = 0.02)	n.a.	n.a.
Valenzise M. et al., 2021 [31]	Italy	12	HC	40	23 (57.5)	C, A	11.6 ± 3.3 *	O	Δ-BMI	30.2 ± 4.0 *	32.0 ± 5.5 *	BMI increase (32 ± 5.5 vs. 30.2 ± 4) not significant	n.a.	N = 38 (95%)	n.a.
Vinker-Shuster M. et al., 2021 [32]	Israel	1	HC	229	117 (51.1)	C, A	0–6 y: N = 60 6–18 y: N = 169	H	aaBWp	38.8 ± 33.7 *	40.4 ± 34.4 *	Overall increase of weight percentile (40.4 vs. 38.8, *p* = 0.03) higher in boys (37.7 vs. 34.4, *p* = 0.01) vs girls (no significant changes), and in patients < 6 yo (39.2 vs. 33.6, *p* = 0.02)	n.a.	n.a.	n.a.
Vogel M. et al., 2021 [33]	Germany	12	HC	274,456	n.a	C, A	6–18	H	ΔBMI-SDS	0.001 (0.001, 0.002) **	0.048 (0.039, 0.056) **	BMI-SDS increase over 3-month AL 1.38 (95% CI 1.30–1.47; *p* < 0.001), >30 times as high as for years 2005–2019. Highest effects in OB group (OR 1.85; 95% CI 1.45–2.35; *p* < 0.001), in all ages	n.a.	n.a.	n.a.
Woolford S. et al. [34]	USA	1	HC	191,509	n.a	C, A	5–17	H	ΔBMI-SD	5–11 y: 0.15 (0.11–0.18) **; 12–15 y: −0.03 (−0.07–0.00) **; 16–17 y: −0.25 (−0.30–−0.21) **	5–11 y: 1.72 (1.67–1.76) **; 12–15 y: 0.87 (0.83– 0.91) **; 16–17 y: 0.23 (0.18–0.28) **	Increase in ΔBMI-SD especially for age 5–11 yo (1.57) vs. 12–15 yo (0.91) vs. 16–17 yo (0.48). OW/OB increase 8.7% (45.7 vs. 36.2%) for age 5–11 yo vs. 5.2% for age 12–15 yo vs. 3.1% for age 16–17 yo	n.a.	n.a.	n.a.

Legend to table: A = adolescents; aaBWp = age-adjusted body weight percentiles; AL = After Lockdown; BL = Before Lockdown; BMI = Body Mass Index (kg/m^2^); BMI-SDS = standardized BMI; BW = Body Weight (kg); C = children d = days; m = months; y = years; CSS = cross sectional study; h = hours; H = hospital; HC = historical cohort; LS = longitudinal study; MFR = muscle to fat ratio; n.a. = not available; NW = normoweight; O = online; OB = obesity; OW = overweight; S = school; w = week; WC = waist circumference; W/H-r = waist/height ratio; yo = years-old; zBW = z-score; Δ = difference from baseline. * = mean ± SDS; ** median and IQR.

**Table 2:** Main patient and study features of articles assessing the risk of hospital admission/ICU in obese children and adolescents included in literature review.

Author	Country	Study Design	Age * (Mean; Range; yr)	N	Males (N, %)	Population	N Obese (%)	Obese Hospitalized/Admitted to ICU (N, %)	Normal Weight Hospitalized/Admitted to ICU (N, %)	Risk Factor and Outcome
Fernandes D.M. et al., 2020 [35]	US	HC; LS	10 (1–17)	250 *	170 (60.5)	C, A	85 (34.0)	85 (100)	165 (100)	Obesity (aOR 3.39, 95% CI 1.26–9.10) severe disease
Graff K. et al., 2021 [36]	US	HC	11 (0–23)	211 *	262 (57.7)	C, A	63 (29.9)	38 (60.3)	21 (14.2)	Obesity (OR 2.48; 95% CI 1.2–5.1), and severe obesity (OR 4.8; CI 1.9–12.1) hospital admission
Guzman et al., 2021 [37]	US	HC	0–21	494	203 (45.6)	C, A	115 (23.3)	36 (31.3)	94 (24.8)	Obesity (ARR 2.02, 95% CI 1.17–3.48) critical illness. Higher risk for age 13–21 yo [ARR 3.09, 95% CI 1.48–6.47]
Kompanyets et al., 2021 [38]	US	CSS	6–18	30,527 *	15.974 (50.2)	C, A	1036 (29.4)	425 (41.0)	2740 (9.3)	Type 1 diabetes (aRR 4.60, 95% CI, 3.91–5.42) and obesity (aRR 3.07, 95% CI, 2.66–3.54) hospitalization
Swann O.V. et al., 2020 [39]	UK	LS	4.6 (0.3–13.7)	602 *	367 (56.0)	C, A	17 (2.8)	17 (100)	585 (100)	age < 1 m (OR 3.21, 95% CI 1.36–7.66), age 10–14 yo (OR 3.23, 95% CI 1.55–6.99), and black ethnicity (OR 2.82, 95% CI 1.41–5.57) admission to critical care
Verma S. et al., 2021 [40]	US	CSS	5 (0.2–15.2)	48 *	52 (63.0)	C, A	19 (39.6)	19 (100)	29 (100)	Obesity ICU admission (63 vs. 28% normal weight, *p* = 0.02)

Legend to table: A = adolescents; ARR = adjusted risk ratio [ARR]; C = children; CI = Confidence Interval; CSS = cross-sectional study; H = hospital; HC = historical cohort; LS = longitudinal study; m = months; S = school; O = online; OR = Odd Ratio; * only participants with known BMI are listed.
